# Rapid monoisotopic cisplatin based barcoding for multiplexed mass cytometry

**DOI:** 10.1038/s41598-017-03610-2

**Published:** 2017-06-19

**Authors:** Ryan L. McCarthy, Duncan H. Mak, Jared K. Burks, Michelle C. Barton

**Affiliations:** 1 Department of Epigenetics and Molecular Carcinogenesis, Center for Cancer Epigenetics, The University of Texas MD Anderson Cancer Center, Houston, TX USA; 2 Department of Leukemia, The University of Texas MD Anderson Cancer Center, Houston, TX USA; 3 Genes and Development Graduate Program, The University of Texas Graduate School of Biomedical Sciences at Houston, Houston, TX USA

## Abstract

Mass cytometry presents an exceptional opportunity to interrogate the biology of highly heterogeneous cell populations, owing to the ability to collect highly parametric proteomic data at a single cell level. However, sample-to-sample variability, due to antibody staining and/or instrument sensitivity, can introduce substantial artifacts into the data, which can in turn lead to erroneous conclusions. This variability can be eliminated by sample barcoding which enables samples to be pooled, stained and run simultaneously. Existing mass cytometry barcoding approaches require time intensive labeling, reduce the number of biologically meaningful parameters and/or rely on expensive reagents. We present an approach utilizing monoisotopic cisplatin to perform cell barcoding that does not require cell permeabilization, can be completed in 10 minutes and can be utilized in combination with existing barcoding techniques to greatly increase the number of samples which can be multiplexed to improve throughput and consistency.

## Introduction

**Figure 1:**
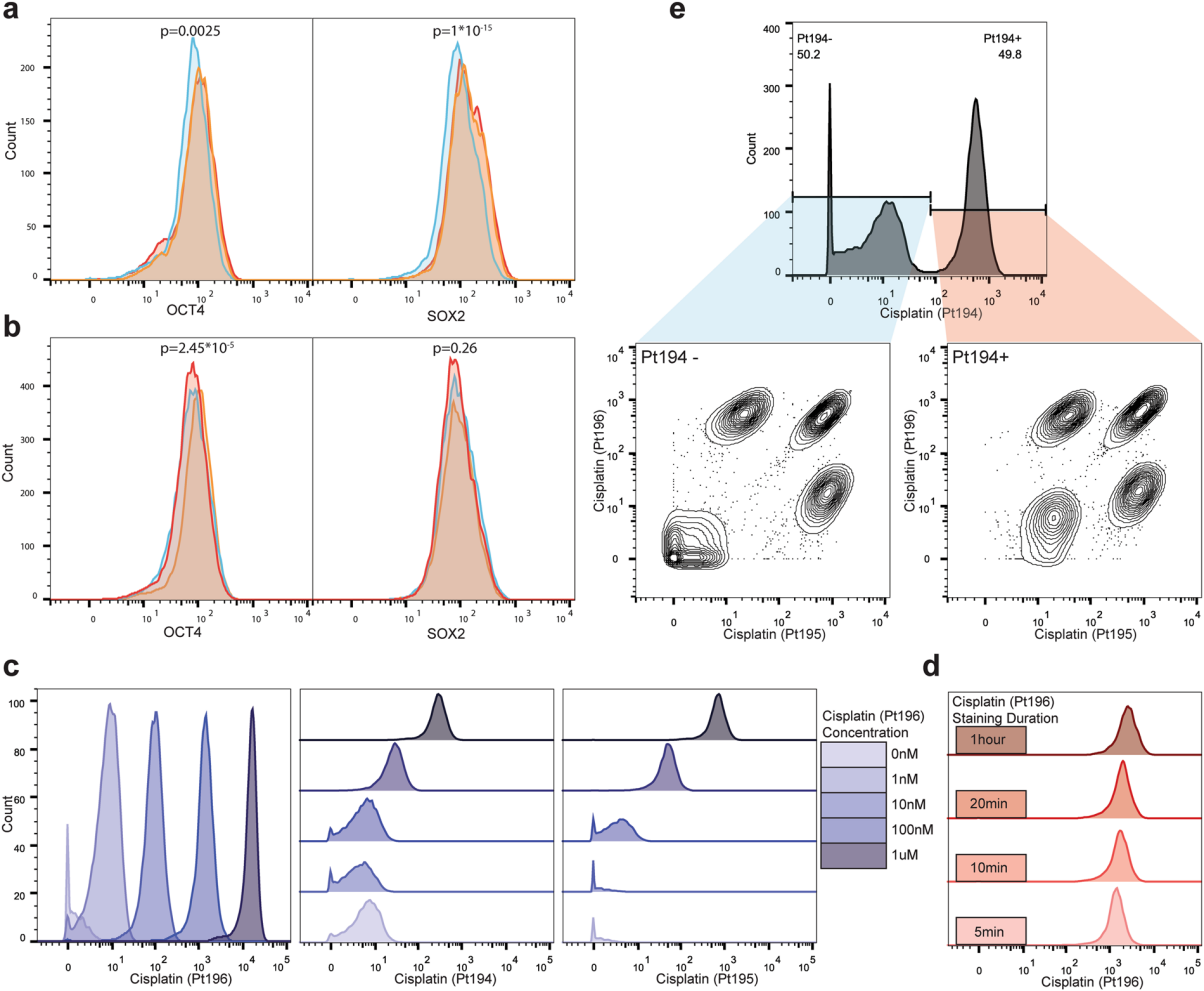
Staining variation between multiple identical samples prepared separately. (**a**) Histograms of a single sample of H9 cells split into three and stained separately following identical protocol. (**b**) Second replicate using a second sample split into three. (**c**) Cells were incubated with the indicated concentrations of cisplatin (Pt196) for 5 min. Signal distributions for the Pt196 channel as well as the measured contributions of the cisplatin Pt196 reagent at the relative concentrations to the Pt194 and Pt195 channels are shown. (**d**) Cells were incubated with 100 nM cisplatin (Pt196) for the indicated time periods. Signal distributions for Pt196 are shown. (**e**) Eight PFA-fixed H9 cell samples labeled with eight combinations of the Pt194, Pt195 and Pt196 cisplatin regents, pooled, and run as a single sample producing eight distinct populations.

Mass cytometry permits the collection of highly parametric data with single cell resolution, enabling the identification of distinct cell states in heterogeneous populations. These highly parametric measurements have great potential for dissecting not only well defined cellular hierarchies but also uncovering previously unexplored heterogeneities of cell states and signaling responses. However, variations during sample processing in antibody concentration, cell number and instrument sensitivity can introduce artifacts creating significant differences between identical cell populations (p < 10^−10^, one-way ANOVA) (Fig. 1a,b; Supplementary Fig. 1a). These problems can be addressed by cell barcoding, which labels all cells in each sample with a unique identifier allowing them to be analyzed as a single multiplexed sample, ensuring consistent antibody labeling between samples, decreasing antibody consumption, and shortening acquisition time.

Previous implementations of cell barcoding for mass cytometry have utilized either direct labeling of cells by chelating lanthanide or palladium isotopes to intracellular components using isothiocyanobenzyl-EDTA^1^ or, alternatively, employing a set of uniquely labeled antibodies against a common target^2^. Lanthanide or palladium chelation methods require cells be transiently^3^ or permanently permeabilized before barcoding, which may disrupt some proteins. Additionally, preparation of chelation reagents is laborious and the labeling process is time intensive, taking 3–4 hours. Alternatively, relatively quick antibody-based barcoding utilizes mass channels for barcoding which could otherwise be used for biologically meaningful parameters. Antibody-based barcoding requires prior knowledge of the cell population, is biased toward cells possessing the target protein and is affected by cell-to-cell variations in protein abundance. There is a clear need for a simple, rapid and unbiased method for mass cytometry cell barcoding.

Cisplatin, a chemotherapeutic agent, previously employed to specifically label dead cells for mass cytometry^4^ contains a platinum atom which is found as six naturally occurring isotopes with ^194^Pt, ^195^Pt and ^196^Pt representing the three most abundant. Here, we describe methods for cell barcoding utilizing monoisotopic cisplatin reagents which enables rapid eight-fold multiplexing and can be used to enhance existing barcoding methods for multiplexing of up to 512 samples. This methodology provides highly accurate and stable cell barcoding without subtracting from the number of mass channels utilized for antibody based measurements.

To ensure that barcoded cells could be accurately assigned to their sample identity, we aimed to maximize the specific signal while minimizing the contribution to the other platinum mass channels. Fixed and permeabilized H9 human embryonic stem cells (hESCs) were incubated with increasing cisplatin concentrations for 5 minutes (Fig. 1c). Cells labeled with concentrations of monoisotopic cisplatin above 1 nM were easily distinguishable from unlabeled cells; however, high concentrations exhibited substantial contributions to the adjacent mass channels. To determine an optimal incubation time, cells were labeled with 100 nM cisplatin over four durations. While a slight increase in labeling intensity was observed for longer incubation periods, robust labeling was achieved after 5 minutes (Fig. 1d). The effect of cell size on cisplatin staining intensity was assessed using H1 and LNCaP cells, small and large cell size respectively (Supplementary Fig. 2). Larger cells exhibited a slight increase in staining comparable to what has been observed with other barcoding techniques^1^. All further experiments were performed with 100 nM monoisotopic cisplatin for 5 minutes, as this approach exhibited a strong signal with excellent resolution between stained and unstained samples, while limiting contribution between mass channels. Additionally, monoisotopic cisplatin barcoding is compatible with the use of cisplatin as a dead cell stain as the monoisotopic Pt194, Pt195 and Pt196 cisplatins contribute minimally to the Pt198 channel (Supplementary Fig. 3a–c) and low background present in the live cells following live/dead staining does not interfere with barcode resolution (Supplementary Fig. 3d).

**Figure 2:**
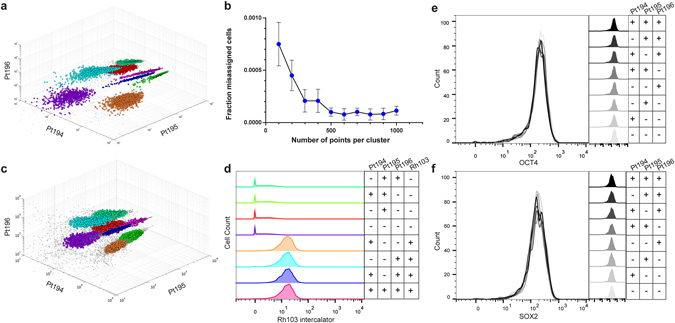
Cisplatin based barcoding uniquely codes eight samples with or without permeabilization eliminating sample-to-sample variability. (**a**) Eight PFA-fixed, methanol-permeabilized H9 cell samples were labeled with eight combinations of the Pt194, Pt195 and Pt196 cisplatin regents, run sequentially, pooled *in silico* and processed by density peak based deconvolution into individual samples as indicated. (**b**) Quantification of misassigned cells resulting from clustering of pooled barcoded data of known sample identity as number of cells per cluster is varied. (**c**) Eight PFA-fixed mouse bone marrow samples were labeled with eight combinations of the Pt194, Pt195 and Pt196 cisplatin regents, half of the samples were labeled with Rh103 intercalator, all samples were pooled, analyzed then separated by density peak based deconvolution into individual samples as indicated. (**d**) Rh103 signal intensity for each cluster identified in (**c**) shown with its corresponding barcode and expected Rh103 status. (**e**,**f**) Histograms, shown overlapping and individually, for OCT4 and SOX2 abundance compared across eight uniquely barcoded H9 cell samples split from the same sample and pooled prior to antibody staining.

It is essential that barcoded, pooled and analyzed cells be distinguished and accurately assigned to their original sample identity. Eight samples of HEK293 cells were fixed with paraformaldehyde (PFA), methanol-permeabilized and barcoded with each of eight potential binary combinations of the three monoisotopic cisplatin reagents. The pooled sample formed eight visually distinct clusters which corresponded in distribution to the anticipated platinum isotope signatures (Fig. 1e). To quantify the accuracy with which sample identity can be assigned based upon the barcode, permeabilized H9 hESC samples with each of the eight possible barcodes were run sequentially and the resulting data was concatenated *in silico*. As existing debarcoding algorithms require all samples to have at least one negative and one positive channel, an assumption which is not satisfied for this dataset, we utilized a density based clustering algorithm^5^ to define the clusters corresponding to each barcode. Clustering of the multiplexed sample identified eight distinct clusters (Fig. 2a). Cells assigned to the resulting clusters were compared to their known sample identity and the percentage of misassigned cells was calculated for a variety of sample cell numbers (Fig. 2b).

We next performed eight sample barcoding on non-permeabilized mouse bone marrow samples to assess i) the effectiveness of cisplatin barcoding on a more heterogeneous primary sample and ii) if robust cisplatin barcoding could be achieved without permeabilization. Mouse bone marrow samples were barcoded, half of the samples were labeled with Rh103 intercalator, and the samples were pooled and analyzed (Supplementary Fig. 4). Clustering of the sample data produced eight distinct cell populations (Fig. 2c). Analysis of Rh103 levels for each cluster demonstrated concordance between samples receiving Rh103 labeling and the clusters exhibiting positive Rh103 staining (Fig. 2d).

To evaluate the effect of cisplatin barcoding on cell sample ratios and staining intensity on samples of varying cell number, spike in experiments were performed where known percentages of HEK293 cell samples were removed, labeled with cisplatin Pt194 and mixed back into the unlabeled cell population. The observed percentage of Pt194 positive cells corresponded to expected ratios (Supplementary Fig. 5a) with the observed, slight decrease in cell number likely due to cell loss during processing. Additionally the signal intensity for the labeled population exhibited minimal varying while varying cell number up to four fold. With low abundance samples, the number of cells may be limited and vary substantially between samples making it desirable to barcode the sample without normalizing the cell numbers. To evaluate the utility of cisplatin barcoding for samples with cell number variation, samples were prepared with varying starting cell numbers. The measured cell number for each cluster matched closely to the known sample cell ratios (Supplementary Fig. 5b) indicating that barcoding does not affect the detection efficiency and performs well even when samples vary in cell number.

The promise of mass cytometry lies in its ability to collect highly parametric data in order to define rare cell populations, dynamic signaling events and unique cell states which can then be compared between multiple samples. The assumption that antibody staining levels are consistent between samples is essential to draw meaningful biological conclusions from mass cytometry data. To determine if antibody staining on pooled barcoded samples was capable of yielding highly consistent staining and highly consistent biological conclusions, we utilized antibodies against OCT4 and p-SMAD2/3 in combination with an existing CyTOF cell cycle panel capable of identifying cells in G0, G1, S, G2 and M-phase (Supplementary Fig. 6)^6^. This panel was chosen since it relies upon antibodies directed against protein epitopes (CyclinB1), phosphorylated proteins (pRb), histone phosphorylations (pH3) and substituted DNA bases (IdU) thus providing a test of the effect of cisplatin barcoding on varied cellular components. Following deconvolution the signal intensity and distribution was compared between each of the barcoded samples (Fig. 2e, Supplementary Fig. 1b). Sample-to-sample antibody staining variation was extremely low with no statistically significant difference between sample means (p > 0.05, One-way ANOVA). Further, quantification of the proportion of cells in each phase of the cell cycle for the deconvoluted samples yielded consistent cell cycle distributions between barcoded samples (Supplementary Fig. 7). These findings illustrate the consistency between samples that is maintained and needed to draw biological conclusions about sample-to-sample differences and diminish the possibility of data artifacts.

Mass cytometry enables routine collection of highly parametric data permitting the discovery of unique cell populations and signaling events which were previously indistinguishable at lower dimensionality. Inconsistency in sample processing, and resultant sample-to-sample variation in antibody staining, may introduce artificial differences that may be misinterpreted as biologically relevant (Fig. 1a). The current study aimed to minimize the potential variability of inter-sample antibody staining by a simple protocol using monoisotopic cisplatin reagents. This approach enables eight-fold multiplexing of samples prior to permeabilization and antibody staining, ensuring that observed differences are due to changes in target proteins and not experimental error, while preserving the sample identity of each cell to a high degree of accuracy.

**Figure 3:**
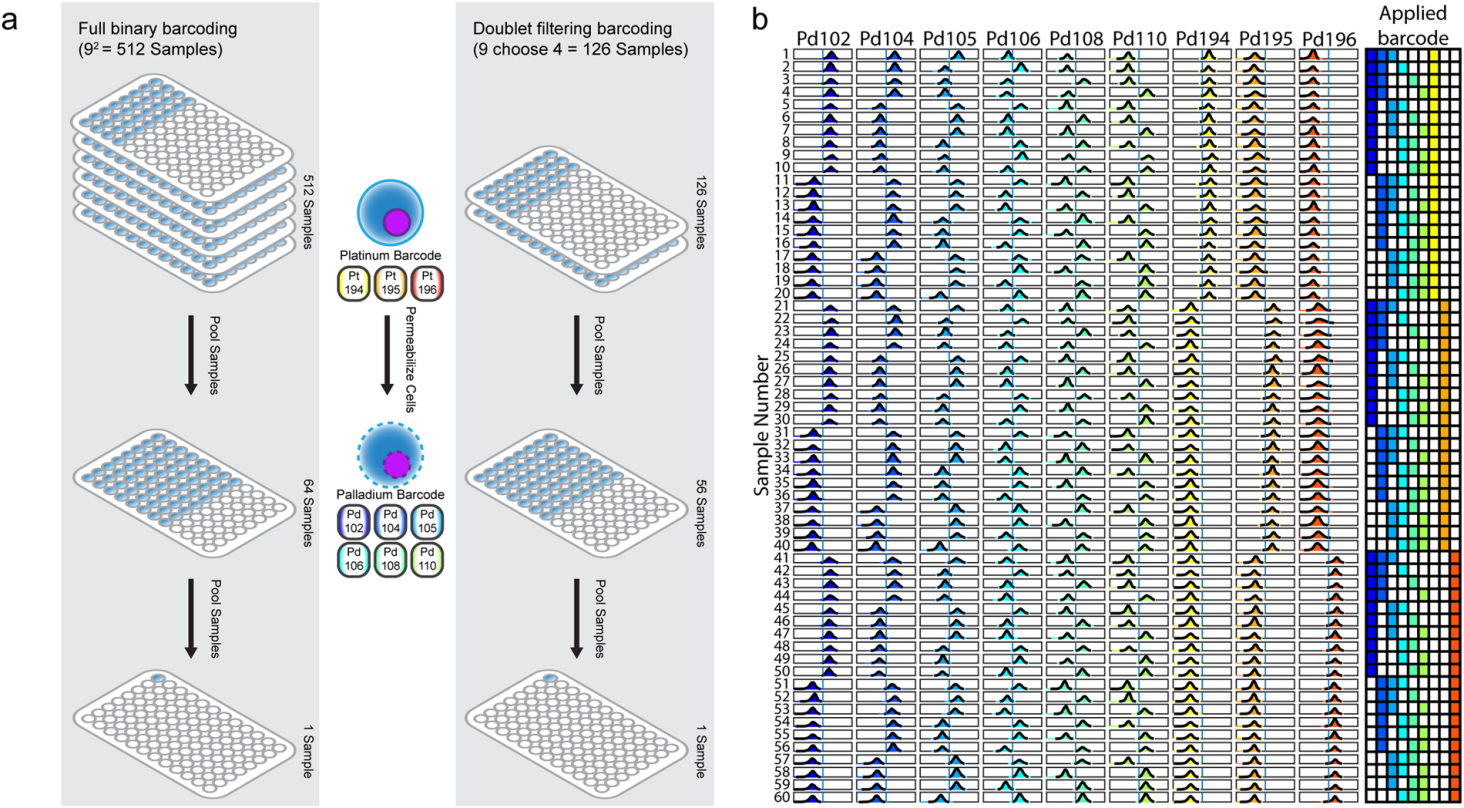
Cisplatin based barcoding used in conjunction with palladium barcoding enables a high degree of CyTOF sample multiplexing. (**a**) Two stage full binary and doublet filtering barcoding schemes utilizing cisplatin in combination with palladium enables a high degree of sample multiplexing. Two proposed barcoding schemes utilizing cisplatin based barcoding early in the sample processing pipeline prior to permeabilization to reduce the effective number of samples being handled and to permit multiplexing of 512 samples or doublet filtering multiplexing of 126 samples when used in conjunction with palladium barcoding. (**b**) Debarcoded 60-plex samples barcoded with a combination of platinum and palladium based barcoding. Each sample possesses a unique barcode that corresponds to the sample specific applied barcode.

Since cisplatin barcoding can be applied to non-permeabilized cells, it may be carried out early in the workflow of sample processing to decrease the number of individual samples being handled and increase throughput. When utilized in combination with six isotope palladium barcoding^1^, monoisotopic cisplatin barcoding can increase sample multiplexing from 64 (2^6^) to 512 (2^9^) samples in a full binary scheme or from 20 (6 choose 3) to 126 (9 choose 4) in a doublet filtering barcoding scheme with minimal additional time and cost (Fig. 3a). To test the compatibility of cisplatin and palladium barcoding we first barcoded 20 samples each with either Pt194, Pt195 or Pt196 then pooled uniquely barcoded samples in groups of three to result in 20 3-plexed samples. Each of these samples was then barcoded with a unique palladium barcode and all samples were pooled (Supplementary Fig. 8). The 60-plex pool was debarcoded into 60 samples by a single cell debarcoding algorithm^1^ and the resulting debarcoded samples exhibited staining patterns corresponding to each of the applied barcodes (Fig. 3b).

For large scale studies such as those comparing hundreds of patient samples it is essential to maintain uniform labeling across all samples to ensure data quality. Our technique for mass cytometry sample barcoding expands the ability to multiplex mass cytometry samples to a critical point which enables improved control of variability for large studies.

## Methods

### Cell Culture

H9 (WA09) human embryonic stem cells (hESCs) were cultured under feeder-free conditions in mTESR1 media (Stem Cell Technologies) on hESC qualified matrigel (BD biosciences) treated plates and passaged every 5–7 days using Dispase (Stem Cell Technologies). HEK293 cells were cultured in DMEM media (Sigma-Aldrich) with 10% fetal bovine serum (Gemini). KBM5 and LNCaP cells were cultured in RPMI 1640 (Corning) with 10% fetal bovine serum. All cell lines were maintained at 37 °C in 4% CO_2_. To obtain single cell suspensions H9 cells were washed with PBS then disassociated with Accutase (Stem Cell Technologies) for 5 min at 37 °C; HEK293 cells were washed with PBS then disassociated with 0.05% Trypsin (Sigma-Aldrich). Following disassociation, H9, HEK293 and KBM5 cells were washed once with serum free DMEM/F12 or DMEM respectively then incubated in serum free media containing 10 µM 5-Iodouridine (Sigma-Aldrich) at 37 °C for 10 min. Live/dead cisplatin staining was done according to the methods from ref. 4.Cells were washed twice with 1%BSA (Equitech Bio) in PBS and once with PBS. Cells were fixed with 2% paraformaldehyde (PFA) (Sigma-Aldrich) pH 6.9 at room temperature for 15 min. Following fixation, 1 × 10^6^ cells/mL were permeabilized with 90% methanol overnight at 4 °C and transferred to −80 °C.

### Bone Marrow Sample Processing

Mice were euthanized and murine bone marrow samples were harvested from C57BL/6 mice and processed to a single cell suspension^7^ following protocols approved by the IACUC of University of Texas MD Anderson Cancer Center. Live/dead cisplatin staining was performed^4^. Cells were washed twice with 1% BSA in PBS and once with PBS. Cells were fixed with 2% paraformaldehyde (PFA), pH 6.9, at room temperature for 15 minutes. Cells were washed and stored at 4 °C in 0.1%PFA and run within 1 week. All animals were treated humanely and according to the guidelines by the IACUC of University of Texas MD Anderson Cancer Center.

### Monoisotopic cisplatin barcoding

Following permeabilization, cells were centrifuged at 300 × g for 5 minutes to remove methanol, then washed with 1 mL of 1% BSA in PBS for each mL of methanol used for permeabilization. Cells were washed once with Wash Buffer (0.5% BSA and 0.02% sodium azide in PBS) and once with PBS. Monoisotopic cisplatin reagents Cell-ID Cisplatin-194Pt, Cell-ID Cisplatin-195Pt and Cell-ID Cisplatin-196Pt (Fluidigm) were stored at 4 °C, as a stock solution of 1 mM in DMSO (Sigma). On the day of each experiment fresh working solutions of the seven possible monoisotopic cisplatin combinations, each monoisotopic cisplatin at a concentration of 200 nM, were prepared in PBS immediately before staining. Cells were resuspended to a concentration of 4 * 10^6^ cells/ml in PBS and monoisotopic cisplatin barcoding was performed by adding an equal volume of the appropriate working stock to the cells and incubating 5 min at room temperature on an orbital rocker. Following incubation, the reaction was quenched with 1 ml of 1% BSA in PBS.

### Palladium barcoding

Palladium barcoding was performed using a Cell-ID 20-plex Pd barcoding Kit (Fluidigm) according to the manufacturer’s instructions. For combined palladium and platinum barcoding the platinum barcoding was performed first, tubes were pooled and the palladium barcoding was performed on the pooled samples.

### Antibody Staining

Following Monoisotopic cisplatin labeling, barcoded samples were washed twice with Wash Buffer, pooled and incubated with the applicable antibodies at a concentration of 4 × 10^5^ cells/µl except where noted otherwise. A complete list of antibodies can be found in supplementary table 1. After antibody staining cells were washed twice with Wash Buffer, once with PBS, then fixed in 0.5 mL 4% PFA in PBS at room temp for 30 minutes. Except where noted otherwise, cells were stained for 10 minutes in 1 mL of either 1:2,000 ^191/193^Iridium (Ir) DNA intercalator (Fluidigm), 62.5 nM final, or 1:1,000 of ^103^Rhodium (Rh) DNA intercalator (Fluidigm), 0.5 uM final, in 4% PFA in PBS. Cells were washed three times with Wash Buffer and analyzed the same day.

### Mass Cytometry Measurement

Cells were analyzed using a CyTOF 2 mass cytometer (Fluidigm). Samples and EQ Four Element Calibration Beads (Fluidigm) were added to a 96-well deep-well plate for autosampler running. Buffer was added just prior to injection where cells were resuspended at a concentration of 5 × 10^5^ cells/ml and run at 45 ul/min. Data were normalized based on bead passport using CyTOF software (v6.0.626, Fluidigm).

### Data Analysis and Visualization

Initial data processing and gating were performed using FlowJo vX10.0. Beads were gated off of the samples and data were gated on singlets, based upon Ir193 and Event Length parameters. Dead cells were removed by either the Pt198 or Rh103 channel. One-way ANOVA was performed in MATLAB (Mathworks) using the anova1 function.

### Barcode Deconvolution

Debarcoding of 60-plex doublet free barcoded data was performed using the single cell debarcoding algorithm^1^ in Matlab (r2015b).

## Electronic supplementary material

Supplementary Information
