# Supplementary material for: Rapid monoisotopic cisplatin based barcoding for multiplexed mass cytometry
Source: Sci Rep. 2017 Jun 19;7:3779. doi: 10.1038/s41598-017-03610-2 (PMC5476666; doi:10.1038/s41598-017-03610-2)
Supplement: Supplementary file 1 — Supplementary Information [file 41598_2017_3610_MOESM1_ESM.pdf]

Rapid monoisotopic cisplatin based barcoding for multiplexed mass cytometry

Ryan L. McCarthy[1], Duncan H. Mak[2], Jared K. Burks[2], Michelle C. Barton[1,3]

[1]Department of Epigenetics and Molecular Carcinogenesis, Center for Cancer Epigenetics, The University of Texas MD Anderson Cancer Center, Houston, TX, USA; [2]Department of Leukemia, The University of Texas MD Anderson Cancer Center, Houston, TX, USA; [3]Genes and Development Graduate Program, The University of Texas Graduate School of Biomedical Sciences at Houston, Houston, TX, USA

Corresponding author: Ryan L. McCarthy, Department of Epigenetics and Molecular Carcinogenesis, Unit 1011, The University of Texas MD Anderson Cancer Center, 1515 Holcombe Blvd, Houston, TX 77030, USA; phone: 713-834-6268; email: rlmccarthy@mdanderson.org

SUPPLEMENTARY FIGURES

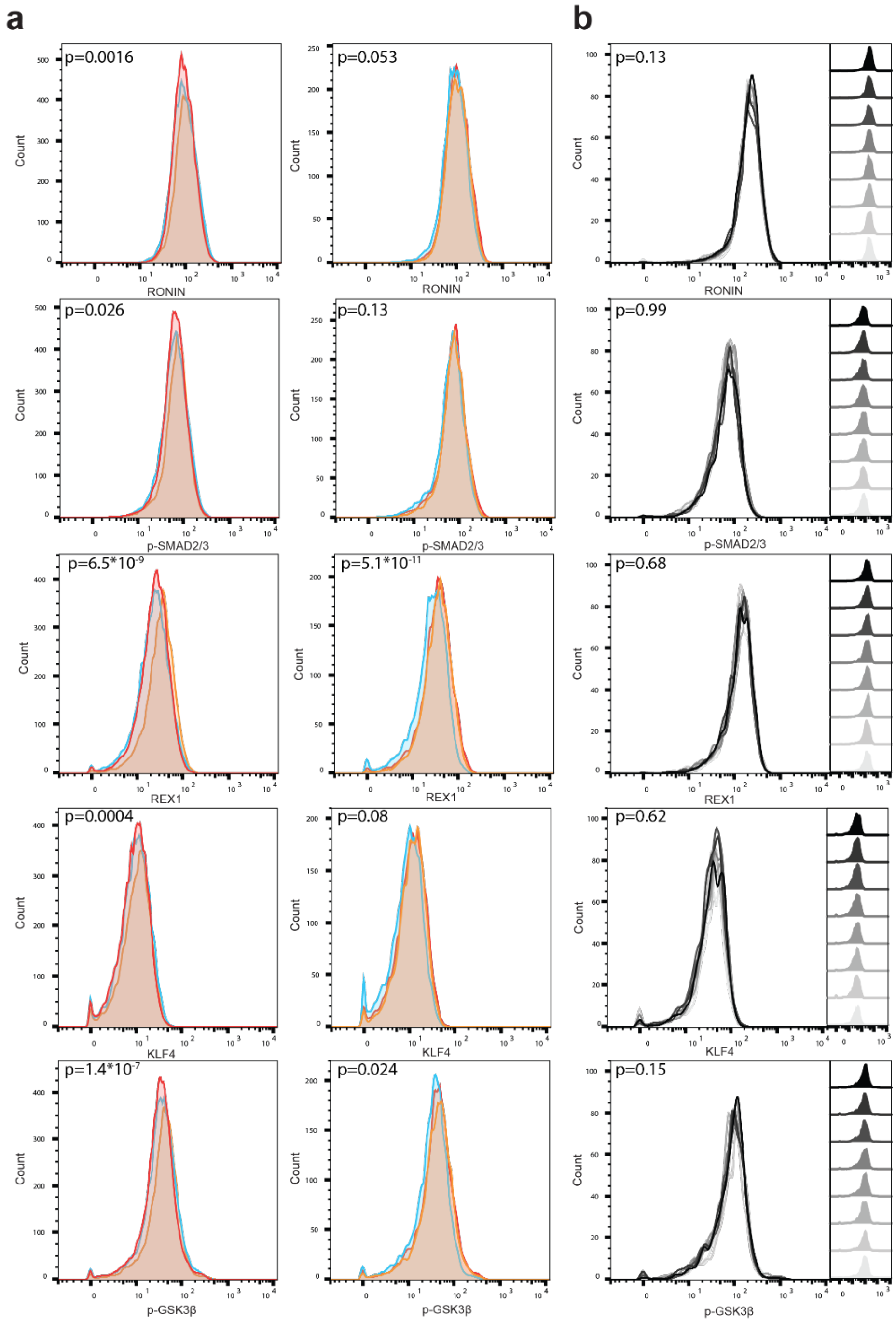

Supplemental Figure 1 | Staining variation between identical samples is corrected by barcoding. **(a)** Two replicates of a single sample split into three and processes separately according to identical protocols showing additional channels to those shown in Figure 1. Statistically significant and not statistically significant at the 0.05 level p-values were observed (one-way ANOVA). **(b)** Histograms, shown overlapping and individually compared across eight uniquely barcoded H9 cell samples split from the same sample and pooled prior to antibody staining. No channel exhibited a statistically significant difference among samples (one-way ANOVA).

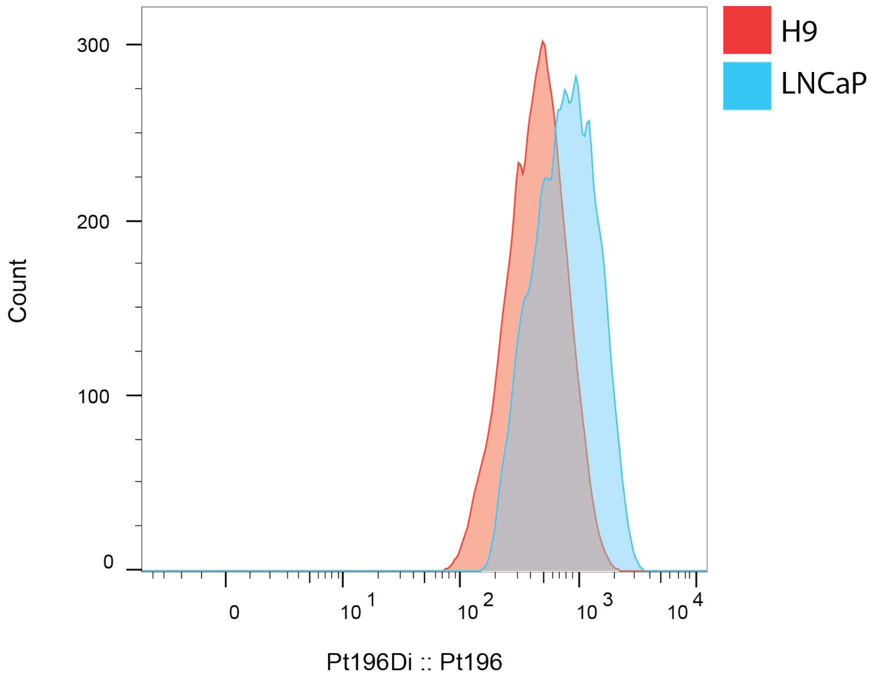

Supplemental Figure 2 | Comparison of cisplatin staining intensity on small (H9) and large (LNCaP) cells. Cells were barcoded, pooled, then stained with monoisotopic cisplatin Pt196.

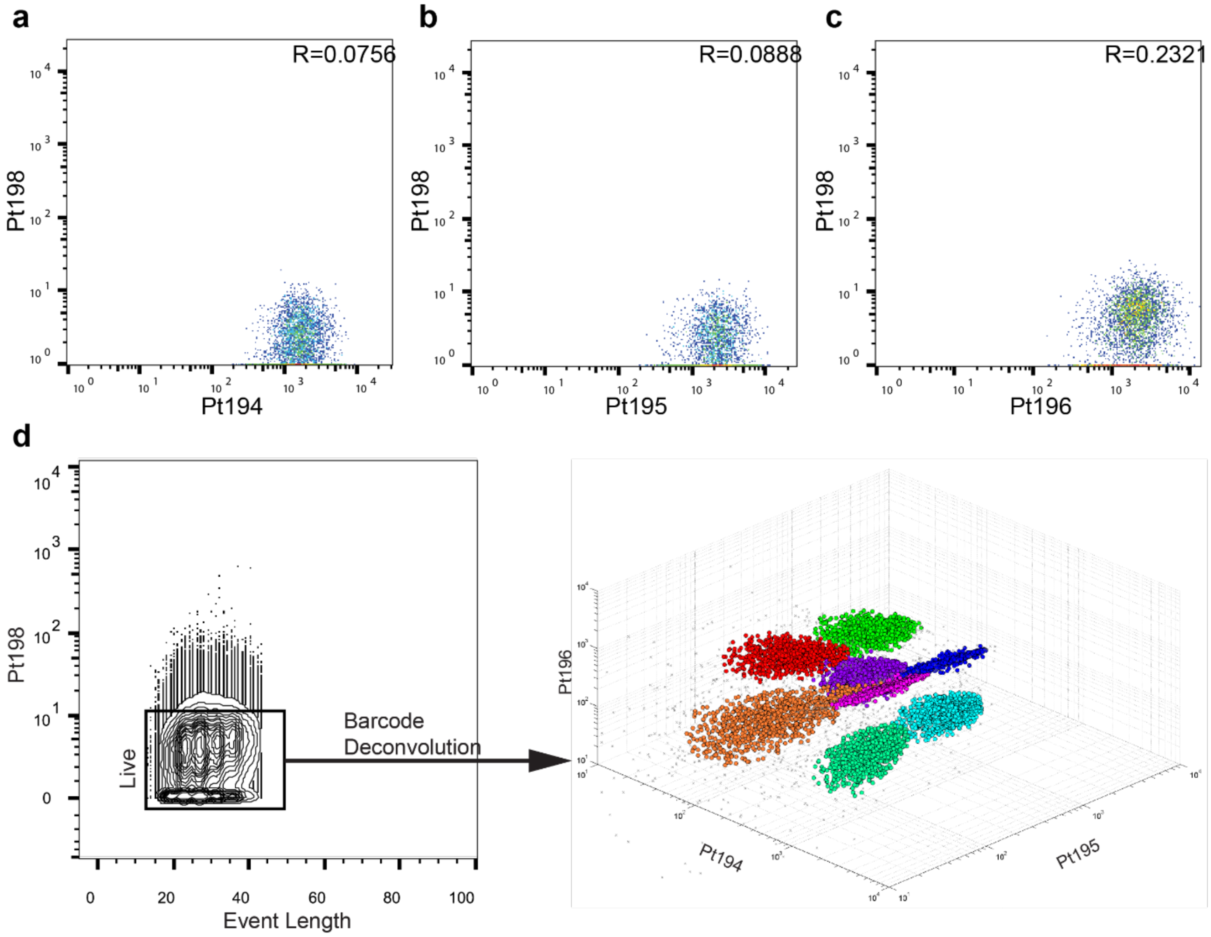

Supplemental Figure 3 | Live/Dead cell discrimination using cisplatin containing the naturally occurring ratio of platinum isotopes is compatible with monoisotopic cisplatin based barcoding. Cross channel contribution to the Pt198 channel in cells labeled with either **(a)** Pt194, **(b)** Pt195 or **(c)** Pt196. **(d)** Live KBM5 cells were identified as the cell population exhibiting low signal in the Pt198 channel. Applying barcode deconvolution to the gated live cells produces eight distinct clusters.

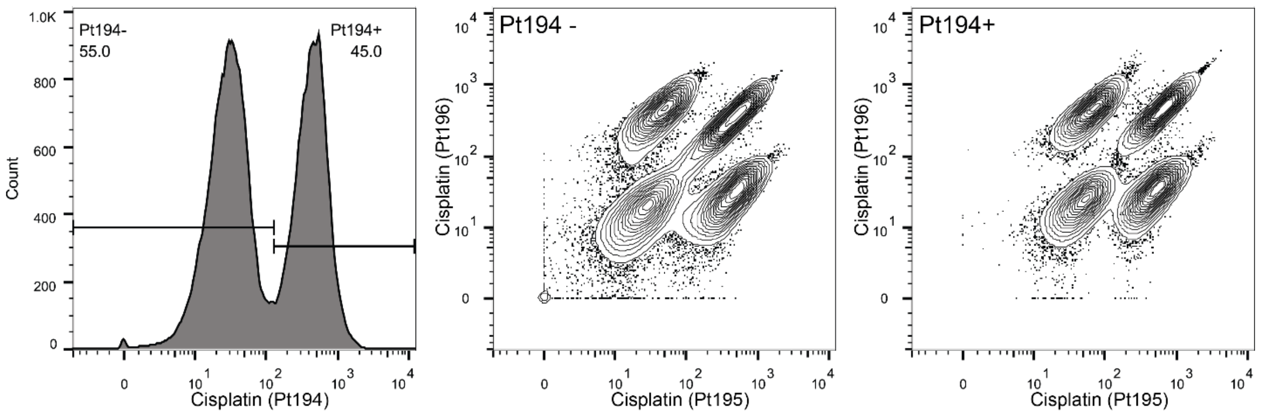

Supplementary Figure 4 | Combinatorial sample labeling with three isotopically purified cisplatin reagents generates eight unique cell barcodes in non-permeabilized cells. Eight PFA-fixed mouse bone marrow samples were stained for live/dead discrimination with non-monoisotopic cisplatin then labeled with the eight possible combinations of the Pt194, Pt195 and Pt196 cisplatin regents. The samples were pooled, mixed and run as a single sample producing eight distinct populations.

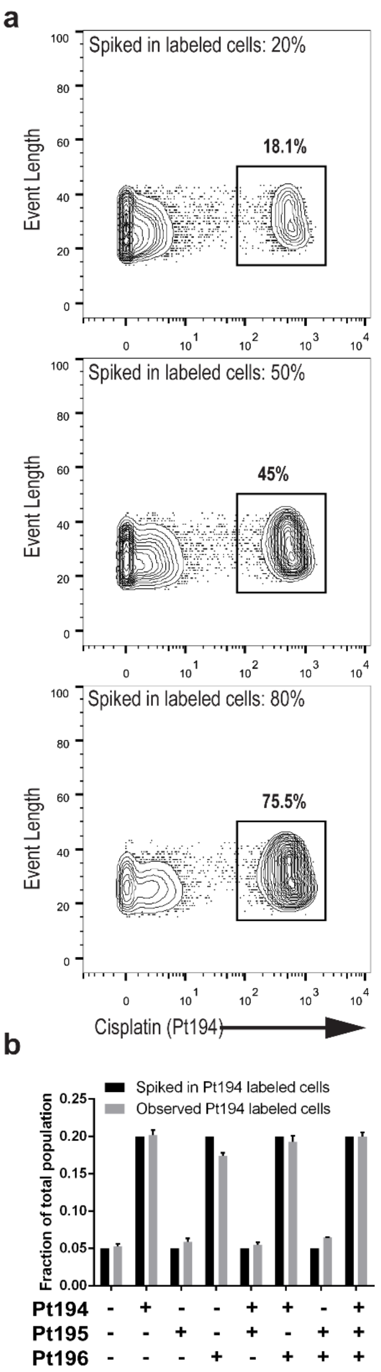

Supplementary Figure 5 | Cell ratios are preserved through barcoding process. **(a)** Three samples of $1*10^6$ starting cells had a known percentage removed, labeled with Cisplatin (Pt194), added back to the sample and analyzed. Percentage of percent total cell population removed for labeling and percent of sample exhibiting Pt194 positive staining are indicated. **(b)** To assess the ability to multiplex samples of varying size, three samples were split into known ratios, labeled with each of the eight barcodes, pooled and analyzed. The expected (black bars) and the observed (grey bars) fraction of total population positive for each barcode are shown with error bars indicating standard deviation.

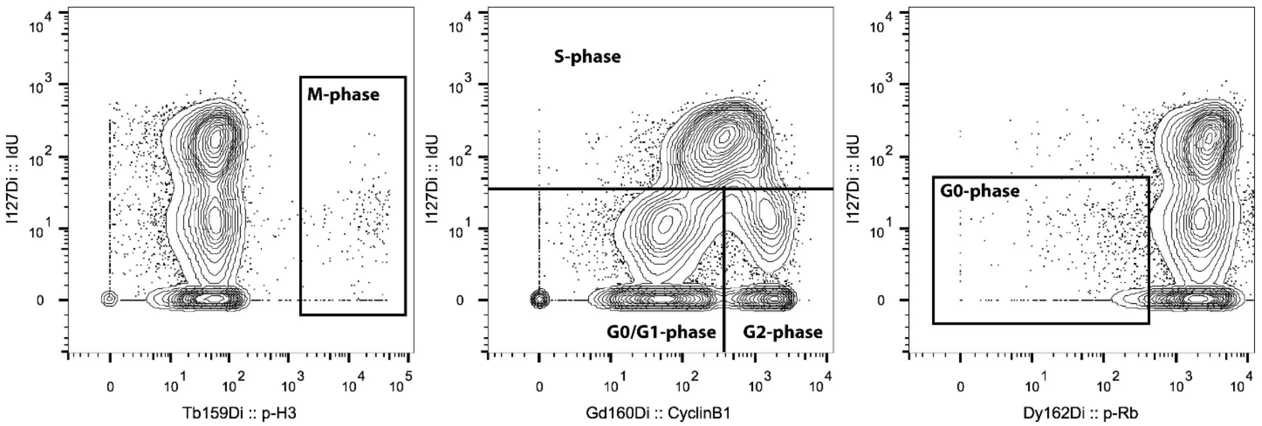

Supplemental Figure 6 | Gating strategy for quantifying percentage of cell population in each phase of the cell cycle.

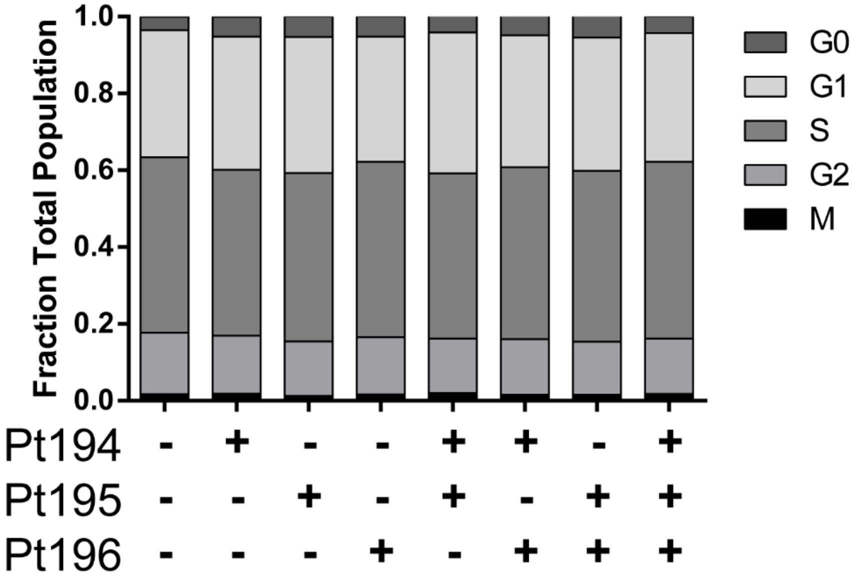

Supplemental Figure 7 | Cell cycle distributions, calculated as shown in Supplemental Figure 5, of the eight uniquely barcoded H9 cell samples from figure 2e. H9 hESCs from a single sample were collected, labeled with 5-iodo-2-deoxyuridin (IdU), which incorporates into replicating DNA to label S-phase cells, and then barcoded, pooled and incubated with antibodies to detect Cyclin B1, p-H3 (Ser28) and p-Rb (Ser807/811).

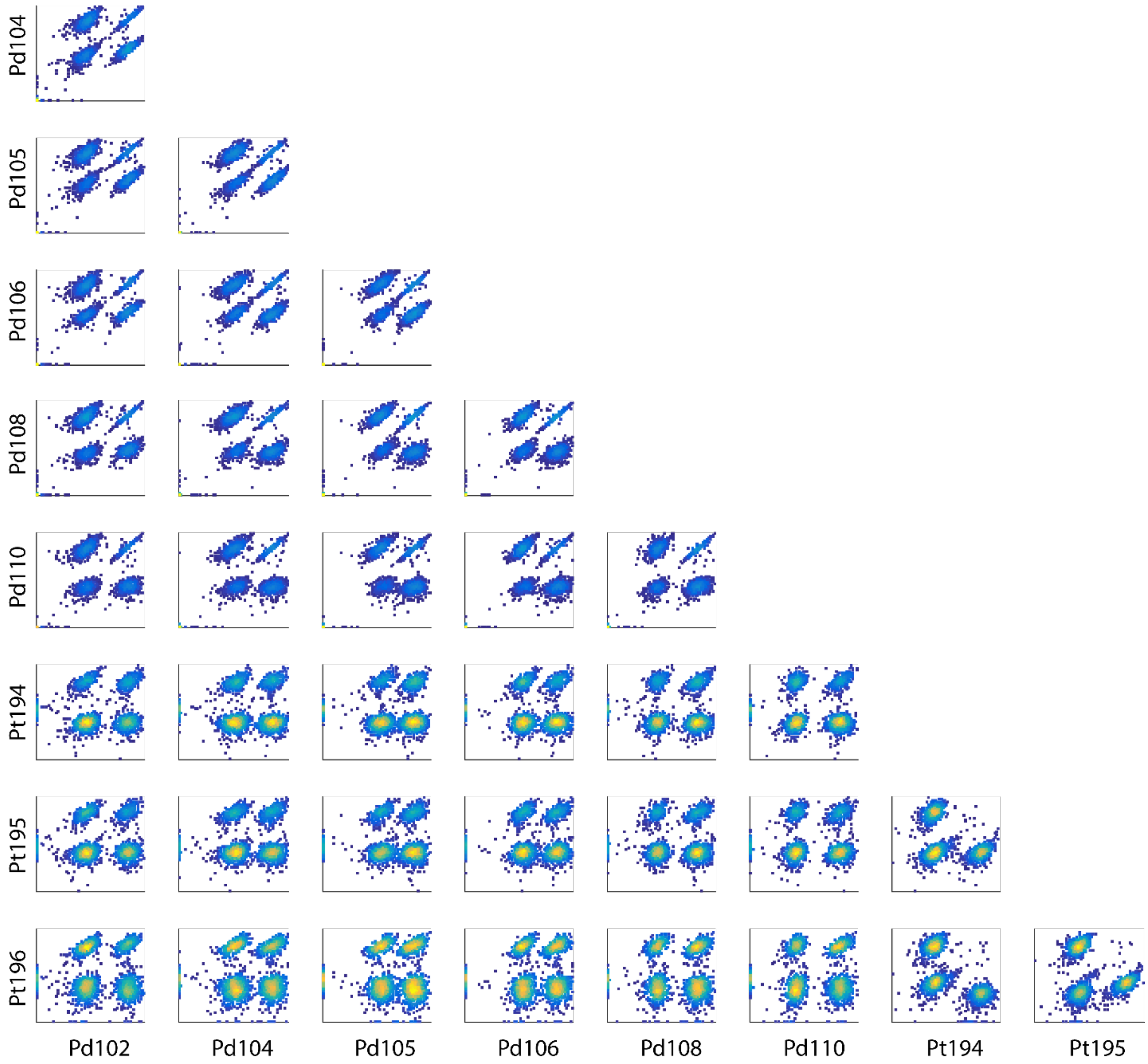

Supplemental Figure 8 | Biaxial plots of all pairs of six palladium and three platinum channels from doublet free barcoding of 60 samples.

Supplemental Table 1 | Antibodies used in this study.

Target	Clone	Conjugated metal tag	Supplier
p-Rb(S807/811)	J112-906	162Dy	BD Biosciences
p-H3(S28)	HTA28	159Tb	Biolegend
Cyclin B1	GNS-1	160Gd	BD Biosciences
Oct4	C-10	146Nd	Santa Cruz
p-Smad2 (S465/467)/3(S423/425)	D27F4	172Yb	Cell Signaling
